# Comparative Analysis of Microstructure and Properties of Wear-Resistant Structural Steels

**DOI:** 10.3390/ma18174002

**Published:** 2025-08-27

**Authors:** Helena Lukšić, Tomislav Rodinger, Vera Rede, Zrinka Švagelj, Danko Ćorić

**Affiliations:** Department of Materials, Faculty of Mechanical Engineering and Naval Architecture, University of Zagreb, 10000 Zagreb, Croatia; helena.luksic@fsb.unizg.hr (H.L.); tomislav.rodinger@fsb.unizg.hr (T.R.); vera.rede@fsb.unizg.hr (V.R.); zrinka.svagelj@fsb.unizg.hr (Z.Š.)

**Keywords:** abrasive wear resistance, microstructure, hardness, Hardox 450, XAR 450

## Abstract

This paper presents the results of wear tests of two types of commercial low-carbon, low-alloy martensitic abrasion-resistant steels, Hardox 450 and XAR 450, which belong to the hardness class 450 HBW. These steels, due to their increased resistance to the abrasive wear mechanism, are used for machine parts for applications in intensive abrasion environments such as construction, mining, and agriculture. The scope of work included microstructure analysis on an optical microscope, chemical composition analysis, Vickers hardness measurements at different loads (HV0.2, HV1 and HV2), and wear testing. Wear tests were carried out by the standard method “dry sand—rubber wheel”, and tests on the Taber abrader device. Microstructure analysis revealed that both steels have a similar non-oriented, homogenous, fine-grained martensitic microstructure. The results of HV2 hardness measurements showed a similar trend for both steels in all examined sections of the plates. For both tested steels, the hardness values of HV0.2 and HV1 are slightly higher than HV2, but the scattering of the results is also greater. Abrasion resistance testing using the standard “dry sand—rubber wheel” method showed that Hardox 450 steel has a lower volume loss of about 8%, but a greater scattering of the results compared to XAR 450 steel. The results of the abrasion resistance test on the Taber abrader device confirmed approximately the same behavior. For both steels, a prediction model was established for a reliable assessment of the wear intensity concerning the grain size. Although examined steels belong to the same hardness class, Hardox steel seems to be a more appropriate choice for the manufacture of machine components exposed to abrasive wear.

## 1. Introduction

Abrasive wear of machine parts is the most common cause of their wear and occurs due to the interaction of two or more parts in relative motion, which leads to degradation of the material surface by separation of wear particles. Degradation of the material surface is manifested by loss of mass and reduction in the thickness of the machine part, as well as changes in the structure and properties of the surface layer [1]. Abrasive wear of structural materials is a common problem in industries such as building construction, automotive, and agriculture, as well as in the mining and processing of mineral raw materials [2,3,4,5,6]. The selection of optimal materials for machine parts that are exposed to intense abrasive wear during operation mainly depends on the following properties: high hardness, favorable toughness and high yield strength, and good weldability [7].

The materials most commonly used for machine parts exposed to intense abrasive wear include martensitic steels, chromium cast iron hardfacing materials, and Hadfield steel [8,9,10,11]. The group of martensitic steels also includes commercial steels with improved resistance to the abrasion wear mechanism [9]. These steels have a precisely balanced chemical composition and a precisely defined heat treatment regime to achieve a martensitic microstructure, and consequently high surface hardness [9]. After quenching, the microstructure of these steels is very homogeneous with fine-needle martensite and possibly with the presence of finely dispersed coherent carbides. In addition to martensite, minor proportions of other phases such as ferrite, pearlite, bainite, or retained austenite may also be present in the microstructure. Some of the types of commercial abrasion-resistant steels are Hardox (SSAB), XAR and TBL steels (ThyssenKrupp Steel Europe AG), Dillidur steels (Dillinger Hütte GTS), Durostat (Grobblech GmbH), and Abrazo (TATA Steel Group) [12]. These commercial steel grades are most often divided according to their Brinell hardness (HB) values. Thus, the commercial name is most often followed by an approximate hardness of 400, 450, 500, 550, or 600 HBW (Brinell hardness using a tungsten carbide ball indenter) [13]. The reason for stating the average hardness next to the name of the steel stems from the dependence of wear resistance on hardness [14].

Białobrzeska et al. [15] conducted a research study comparing the wear resistance of commercial steels with improved wear resistance, of the same hardness class, from two different manufacturers. The tested steels were Brinar 400, Brinar 500, Hardox 400, and Hardox 500. Steels with higher hardness showed better wear resistance, which confirms the strong correlation between abrasion resistance on steel hardness [15]. However, steels with the same average hardness can result in different abrasive wear micromechanisms. Haiko et al. [16] studied the influence of hardness and microstructure on abrasion resistance. The research was conducted on steel samples of equal hardness, but different microstructures. It was concluded that for more inhomogeneous microstructures, the coefficient of friction is highly variable and that depending on the microstructure, different micromechanisms of abrasive wear are obtained [16]. Zemlik et al. [8] carried out a comparative analysis of the wear resistance of five different commercial steels (Creusabro 4800, Creusabro 8000, TBL PLUS, XAR 600, 38GSA), with hardness classes between 500 and 600 HBW. They found that the abrasion resistance of the tested steels is very similar even though their hardnesses are different, but they differ in micromechanisms of wear [8]. Wear resistance depends not only on the material hardness but also on many other parameters. One of the most important factors is the type of abrasive. In their research, Szala et al. [17] compared the resistance to the abrasive wear mechanism of five different structural steels (S235, S355, C45, AISI 304, and Hardox 500) with three different abrasives (garnet, corundum, and carborundum). It was found that different abrasives have different effects on the abrasion resistance of the tested steels [17].

The objective of this research was to compare the wear resistance and microstructure of two commercial wear-resistant structural steels (Hardox 450 and XAR 450) which are often used to make machine parts exposed to intense abrasion or harsh environments. To achieve this goal, resistance to the abrasive wear mechanism was tested using the standard “dry sand—rubber wheel” method and on a Taber abrader device. Microstructure analyses were also performed using a light microscope in the etched state, and hardnesses with different loads were measured using the Vickers method. In addition, a statistical analysis was carried out to determine the correlation between wear resistance and material hardness. The outcomes of this study have the capability to provide valuable insights into the selection of wear-resistant steels, taking into account the diverse operational conditions defined with abrasive grain size.

## 2. Materials and Methods

The materials used for this study were Hardox 450 (SSAB) and XAR 450 (ThyssenKrupp Steel Europe AG) in the form of steel plates. The thickness of the Hardox 450 plate was 5 mm, while the thickness of the XAR plate was 4 mm. Test specimens were cut from plates using a water jet to preserve the microstructure obtained by the rolling process.

Chemical composition analysis was performed on sections of both steels with a glow discharge atomic emission spectrometer GDOES 850A (Leco, Saint Joseph, MI, USA) with the following parameters: U = 1200 V, I = 40 mA, 99.999% Ar. The obtained results were the arithmetic average of three measurements taken at different spots on both steels.

In order to calculate the volume loss and wear rate following the wear tests, the density of both materials was measured in accordance with Archimedes’ principle using an analytical balance JP703C (Mettler Toledo, Zürich, Switzerland). According to Archimedes’ principle, density determination is based on measuring the mass of test samples in air and then in a medium of constant temperature. In this study, distilled water at a temperature of 25 °C was used as the medium. The mean density value obtained from three measurements for Hardox 450 steel was 7.648 g/cm^3^, while for XAR 450 steel it was 7.588 g/cm^3^.

**Figure 1:**
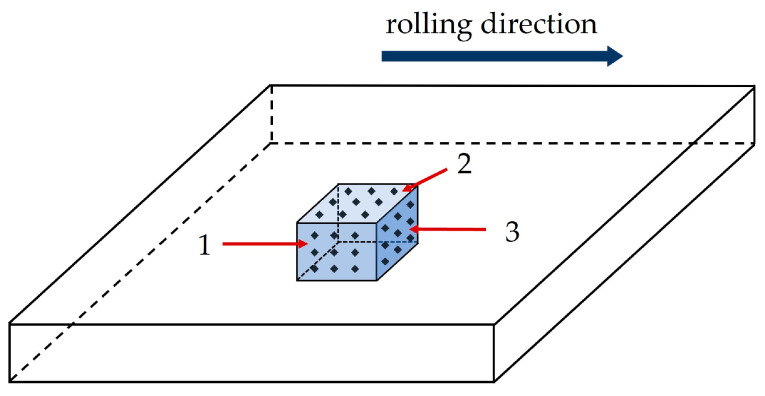
Three characteristic sections considering the plate rolling direction: (1) in the rolling direction and perpendicular to the plate surface; (2) parallel to the plate surface; and (3) perpendicular to the rolling direction and to the plate surface.

Microstructural analysis was performed on three characteristic sections relating to the rolling direction of the plate: (1) in the rolling direction and perpendicular to the plate surface; (2) parallel to the plate surface; and (3) in the transverse direction and perpendicular to the plate surface, as illustrated in Figure 1. Microstructural analysis was performed on an Olympus GX51 (Olympus, Tokyo, Japan) optical microscope, after etching the sample surfaces in a 5 wt.% HNO_3_ solution in ethyl alcohol (Nital).

Hardness measurements were performed on polished surfaces of three characteristic sections of both steels, using the Vickers method with three different loads—1.961 N (HV0.2), 9.807 N (HV1), and 19.61 N (HV2). Hardness measurements of HV0.2 and HV1 were performed on a Tukon 2100B hardness tester (Instron, Norwood, MA, USA), while hardness measurements of HV2 were made on a ZHVµ (ZwickRoell, Ulm, Germany) device. At each characteristic section and at each load, nine hardnesses were measured and the mean value was calculated. The hardness measurement spots are marked in Figure 1.

**Figure 2:**
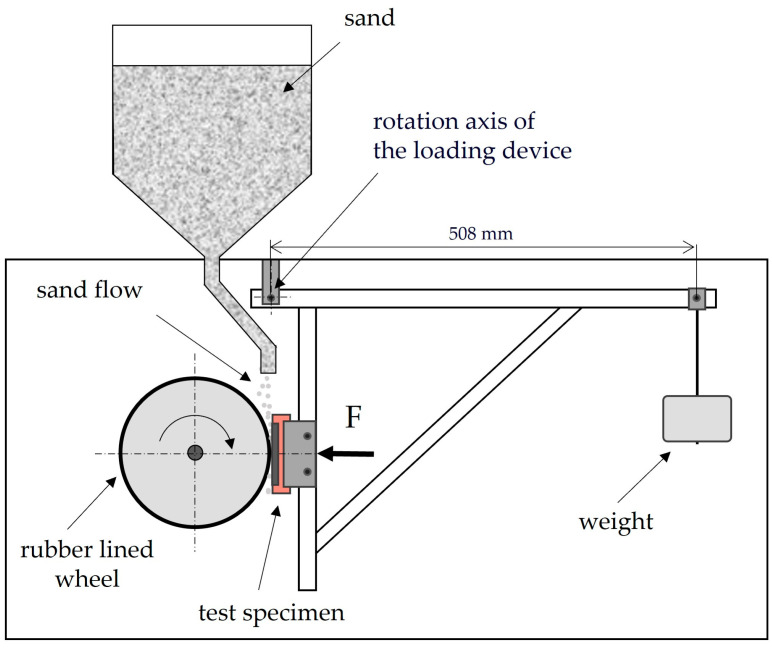
Scheme of a wear testing device by “dry sand—rubber wheel” method.

**Figure 3:**
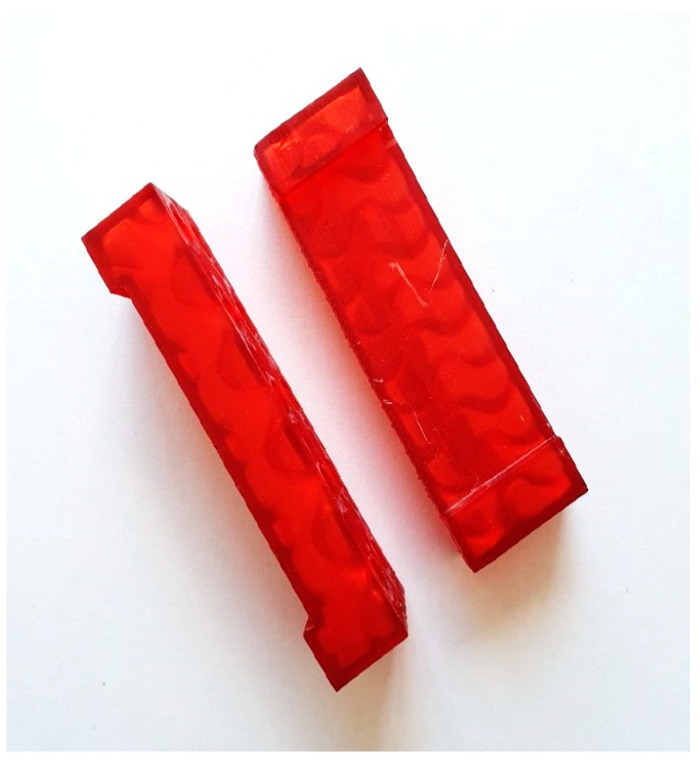
Sample holders made for Hardox 450 and XAR 450 samples.

The abrasive wear test was carried out using the “dry sand—rubber wheel” method in accordance with Procedure B of the ASTM G65-16 (2021) standard, which prescribes a test load of 130 N on the wheel and a total number of wheel revolutions of 2000, giving a total abraded distance of 1436 metres [18]. The wheel rotation speed was 200 rpm, while the peripheral speed was 2.33 m/s. A schematic representation of the “dry sand—rubber wheel” device (Faculty of Mechanical Engineering and Naval Architecture, Croatia) is illustrated in Figure 2. During the test, the surfaces of the samples were abraded with B35S quartz sand (Kema, Puconci, Slovenia). The particle size ranged from 0.063 to 0.355 mm. The mass of the sample was measured on a precision balance B5C 1000 (Mettler Toledo, Zürich, Switzerland) before and after each wear cycle and based on these measurements, the mass loss (Δ*m*) was calculated. Due to the different densities of Hardox 450 and XAR 450 samples, the mass loss was converted into volume loss (Δ*V*). The recommended dimensions of test samples according to the ASTM G65 standard are 12 mm × 25 mm × 75 mm. The samples for this study were cut from Hardox 450 and XAR 450 steel plates, and due to the thickness of the plates, the cut samples had dimensions of 5 mm × 25 mm × 60 mm and 4 mm × 25 mm × 60 mm, respectively. Due to their slightly smaller thickness, two sample holders were made, which allow for proper positioning of the sample on the wheel with respect to the direction of load and thus the implementation of a valid test. Polymer sample holders were made by additive manufacturing (Figure 3). For both steels, tests were conducted on 5 samples, on both front and back surfaces with dimensions of 25 mm × 60 mm, making a total of 10 measurements.

**Figure 4:**
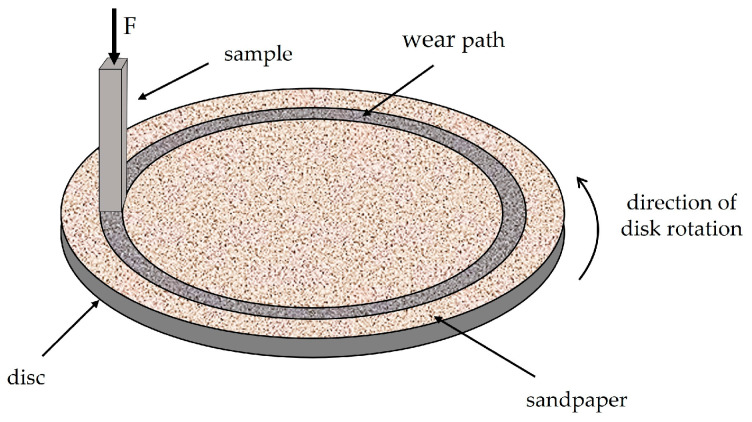
Schematic illustration of tests on the Taber abrader device.

The wear resistance test on the Taber abrader device (Taber Industries, North Tonawanda, NY, USA) was performed on samples that were in the form of a four-sided prism with dimensions of approximately 4 mm × 5 mm × 30 mm (Hardox 450) and 4 mm × 4 mm × 30 mm (XAR 450). During the test, surfaces parallel to the rolling direction and perpendicular to the plate surface were worn, with dimensions of 4 mm × 5 mm for Hardox 450 steel and 4 mm × 4 mm for XAR 450 steel. The Taber abrader consists of a rotating disc with a diameter of 125 mm rotating at a speed of 60 rpm, on which sandpaper is placed. The constant force with which the sample was pressed against the sandpaper was 4.91 N, while the relative tangential speed of the sample during the test was 0.251 m/s. In this test, five test samples for each steel were tested on five different types of Al_2_O_3_ sandpaper with different grain sizes. The types of sandpapers used in this study were P1200, P800, P500, P400, and P320, corresponding to average abrasive grain sizes of 15.3, 21.8, 30.2, 35.0, and 46.2 µm, respectively [19]. The wear test of an individual sample was carried out on each sandpaper for 100 s, which corresponds to a wear length of approximately 25 m. The order of sandpapers used during testing ranged from the finest (P1200) to the coarsest (P320). Before and after each wear cycle, the mass of the samples was measured on an analytical balance B5C 1000 to calculate the mass loss (Δ*m*) and determine the volume loss (Δ*V*). In addition, before each wear cycle, the dimensions of the worn surface were measured to calculate the wear rate (ώ), which represents the ratio of volume loss to worn surface area. A schematic presentation of tests on the Taber abrader device is shown in Figure 4.

The morphology of surfaces subjected to wear testing on a Taber abrader, after wear test with P320 grade sandpaper, was analysed with an EM-30AX Plus (COXEM, Daejeon, Republic of Korea) scanning electron microscope, using backscattered electron (BSE) microscopy with an accelerating voltage of 20 kV.

## 3. Results and Discussion

### 3.1. Chemical Composition Analysis

Table 1 shows a comparison of the chemical composition of Hardox 450 and XAR 450 steels according to the manufacturer’s data (MD) and the results obtained by chemical analysis on a GDOES device (OR) [20,21]. Hardox 450 steel has a slightly higher carbon content than XAR 450 steel; however, both steels belong to the group of low-carbon steels. Manganese is a stabilizer of austenite and in these steels, it enhances hardenability. However, it lowers the temperatures of the start and finish of the martensitic transformation (M_s_ and M_f_ temperatures) [22,23,24]. Small contents of chromium, nickel, molybdenum, and boron were observed in both analysed steels. The final microstructure of wear-resistant steel is primarily influenced by the content of the alloying elements such as boron, molybdenum, and silicon. Higher quantities of molybdenum and silicon lower the martensite start (M_s_) temperature. However, if the martensitic transformation takes place at lower temperatures, distortion of the steel plate may occur [24,25]. In both steels, the mass content of phosphorus and sulfur impurities is within permissible limits and does not negatively affect the mechanical properties of the steel.

### 3.2. Microstructural Analysis

**Figure 5:**
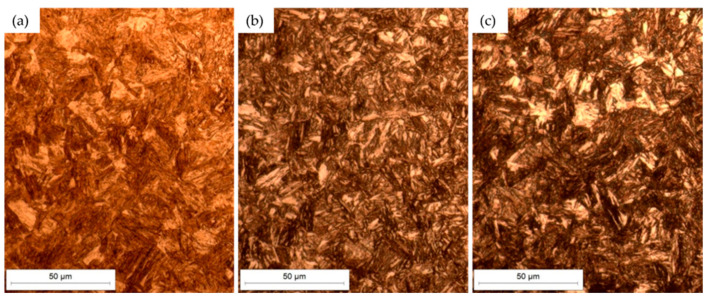
Microstructure of Hardox 450 steel in the etched state: (**a**) longitudinal section; (**b**) parallel to the surface; (**c**) cross-section.

**Figure 6:**
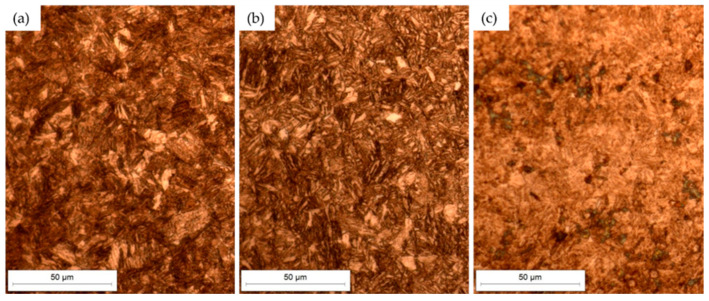
Microstructure of XAR 450 steel in the etched state: (**a**) longitudinal section; (**b**) parallel to the surface; (**c**) cross-section.

Figure 5 and Figure 6 present the microstructures of Hardox 450 and XAR 450 steels in the etched state. The micrographs show the surface microstructure at 500× magnification in three characteristic sections in relation to the rolling direction of the plate: longitudinal section (Figure 5a and Figure 6a); section parallel to the surface (Figure 5b and Figure 6b); and cross-section (Figure 5c and Figure 6c). In both Hardox 450 and XAR 450 steels, the microstructure is similar on all three characteristic sections. Figure 5 and Figure 6 show that both steels have a fine-grained martensitic microstructure, with a varied orientation of martensite needles and plates. No preferred direction of microstructure orientation was observed in any of the steels. Optical microscopy did not detect retained austenite or carbides, which was to be expected due to the low carbon content in these steels. With the aim of conducting a more detailed examination of the microstructure, more advanced microscopic analyses are needed, such as scanning electron microscopy and X-ray diffraction.

**Figure 7:**
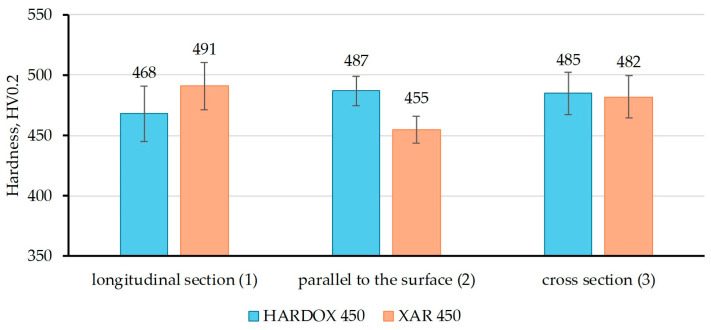
Hardness HV0.2 of Hardox 450 and XAR 450 steels in characteristic sections.

**Figure 8:**
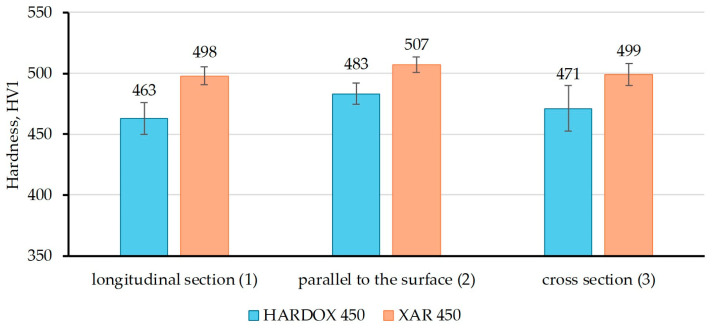
Hardness HV1 of Hardox 450 and XAR 450 steels in characteristic sections.

**Figure 9:**
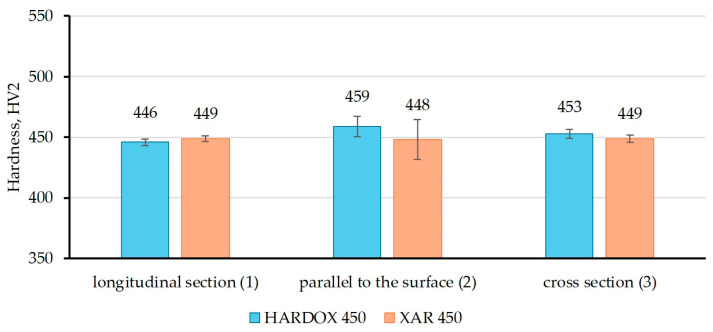
Hardness HV2 of Hardox 450 and XAR 450 steels in characteristic sections.

Figure 7, Figure 8 and Figure 9 show the results of hardness measurements with different loads (HV0.2, HV1, and HV2), at three characteristic sections in relation to the rolling direction of the plate. The figures present the mean values of nine repeated measurements with standard deviation.

From Figure 7, it is evident that in the longitudinal section, the hardness HV0.2 value is higher for XAR 450 steel, while on the sample parallel to the plate surface, the hardness is higher for Hardox 450 steel, and on the cross section, the samples have approximately equal hardness values. The highest HV0.2 value for XAR 450 steel was measured on the longitudinal section in relation to the rolling direction (491 HV0.2), while for Hardox 450, the hardness values of the sample parallel to the surface and the cross-section sample are approximately equal (487 and 485 HV0.2, respectively). Figure 8 shows the results of the HV1 hardness measurements, and it is noticeable that the hardness of XAR 450 steel is higher on all characteristic sections compared to Hardox 450 steel. At this load, the highest hardness values of XAR 450 steel were measured on the surface parallel to the rolled surface (507 HV1), while the longitudinal and transverse sections have slightly lower hardness values (498 and 499 HV1, respectively). Therefore, it is clear that this steel has nearly equal hardness on all three tested sections. While the HV0.2 and HV1 hardnesses of Hardox 450 are equal across the characteristic cross-sections, higher HV1 hardnesses were measured for XAR 450 steel across all three cross-sections tested. This may be due to the slightly smaller grain size of XAR 450 steel, whereby the indentation captures a greater number of grain boundaries, which are generally harder than the grain interior, resulting in higher hardnesses. The HV2 hardnesses give the most uniform values for both steels in all sections. These hardnesses are lower than those measured for the HV1 method due to the application of a higher diamond pyramid indentation load, which corresponds to the classic indentation size effect (ISE) in which the measured hardness of a material decreases as the indentation size increases during Vickers hardness testing at low loads [26,27].

The results of the abrasive wear test of the samples using the “dry sand—rubber wheel” method are shown in Table 2. The test was carried out on the section parallel to the plate surface and repeated ten times for both steels.

**Figure 10:**
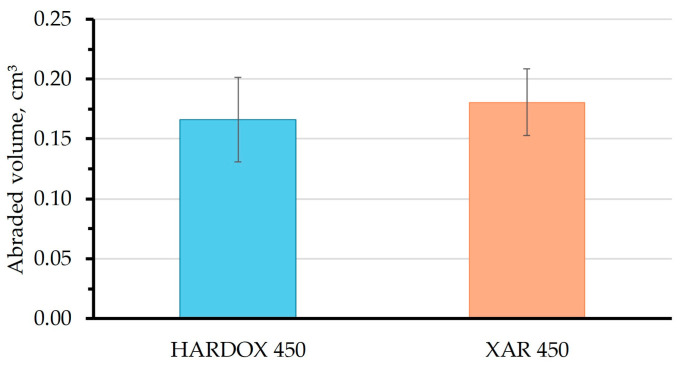
Average abraded volume by the “dry sand—rubber wheel” method.

The true abrasion resistance shall be expressed as volume loss, considering differences in steel density (Figure 10).

From Figure 10, it can be seen that Hardox 450 steel samples had a smaller abraded volume and a higher wear resistance compared with XAR steel samples when tested using this method. This can be attributed to the slightly higher hardness of Hardox 450 steel measured on a section parallel to the surface on which the wear test was conducted.

The results of the mass loss of the samples after wear tests on the Taber abrader device are shown in Table 3. The tests were carried out in order from the finest to the coarsest sandpaper, with surface polishing between each step.

Based on these mass loss values (Δ*m*), since the tested steels have different densities (*ρ*) and the samples have slightly different cross-sectional areas (*A*), the wear intensity (*ώ*) for each sandpaper was calculated using Equation (1):

(1) $$\overset{´}{\omega }=\frac{∆m}{\rho \cdot A},\;{\mathrm{mm}}^{3}/{\mathrm{mm}}^{2}$$

**Figure 11:**
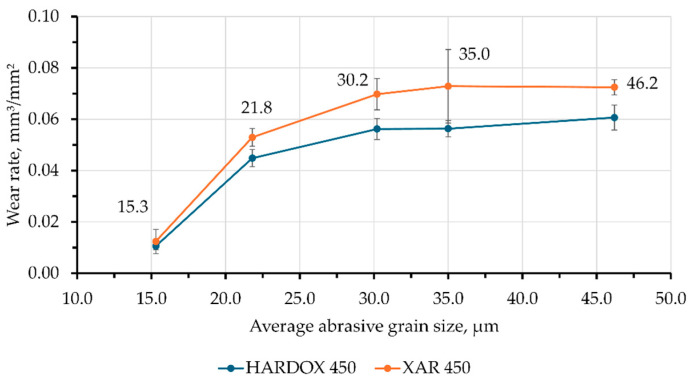
Wear intensity of samples on the Taber abrader device.

The calculated wear intensity values of the tested steels are shown in Figure 11.

The diagram in Figure 11 clearly demonstrates the correlation between the average abrasive grain size and the wear intensity for both materials, with the greater wear resistance of Hardox 450 steel being clearly visible, as was also the case with the “dry sand—rubber wheel” test. The wear intensity of Hardox 450 steel is 15–25% lower than the other tested steel, for all tested sandpapers. For both steels, a characteristic trend is observed where the wear intensity strongly increases with increasing abrasive grain size, but only up to a certain critical size, which for these steels is between 21.8 and 30.2 μm. After that, the increase in wear intensity becomes milder or is even constant, indicating that this is the critical abrasive grain size for the tested steels. This phenomenon, in which the wear rate trend changes with increasing abrasive grain size, is known as the critical abrasive grain size. It has been reported in various metallic [28,29,30,31,32,33] and non-metallic [34] technical materials. Hardox 450 shows a lower wear intensity in all conditions, and the standard deviations are relatively small, which indicates a homogeneity of the microstructure and consistency of tribological properties. For XAR 450 steel, a slightly higher wear intensity was observed for all grades of sandpapers, and for certain sizes of abrasive grains, there is a greater dispersion of the results. The greater scatter of results in the case of XAR 450 steel probably results from more heterogeneous microstructure and uneven tribological behavior on the surface parallel to the rolling direction and perpendicular to the plate surface (longitudinal section) that was worn on the Taber abrader device.

These results are somewhat unexpected given the higher hardness values of XAR 450 steel. However, it is a known and generally accepted opinion that higher hardness values do not necessarily mean higher wear resistance. In addition to hardness, material toughness and crack propagation resistance have a great influence on the intensity of abrasive wear.

**Figure 12:**
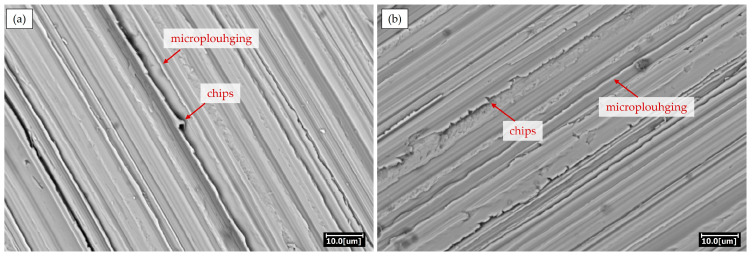
Morphology of surfaces subjected to wear testing on the Taber abrader device, after sandpaper P320: (**a**) Hardox 450; (**b**) XAR 450.

Analysis of the abraded surfaces of the examined steels revealed that microploughing was the dominant wear mechanism for both steels (Figure 12). In XAR 450 steel, there are significantly more chips at the groove edges than in Hardox 450 steel, indicating that XAR 450 is more prone to the formation and propagation of cracks at the groove edges, which is the reason for the higher volume loss during abrasion. The amount of chips at the groove edges can be explained by the toughness values of these steels. According to the manufacturer, Hardox 450 steel has a higher Charpy V impact energy value (guaranteed 50 J/−40 °C [20]) than XAR 450 steel (unguaranteed 40 J/−40 °C [21]).

Based on the wear intensity values, an analysis of variance (ANOVA) was also performed using second-order polynomial regression, where the average sizes of abrasive grains and their squares were used as input variables. The analysis was performed using Microsoft Excel, and the results for Hardox 450 steel are shown in Table 4 and for XAR 450 steel in Table 5.

Based on the second-order regression analysis (Table 4 and Table 5), it is noticed that the test results describe the behavior of the material well under abrasive wear conditions by the Taber abrader. The results describe the behavior of Hardox 450 steel somewhat better (coefficient of determination R^2^ = 0.91158), while for the XAR 450 sample the coefficient of determination is somewhat lower, but still describes the behavior of this steel very well during wear (R^2^ = 0.89360), which is confirmed by the significance F value, which is very low for both regressions (lower than 10^−10^, while statistically significant models are those for which this value is less than 0.05). From the results shown in Table 4 and Table 5, equations can be derived that describe the wear intensity of individual steels. For Hardox 450 steel, this equation is:

*ώ*_HARDOX_ = −0.06787 + 0.00669 ∙ *X* − 8.548 ∙ 10^−5^ ∙ *X*^2^, (2)

while for XAR 450 steel:

*ώ*_XAR_ = −0.09457 + 0.00902 ∙ *X −* 1.177 ∙ 10^−4^ ∙ *X*^2^, (3)

where:

*ώ*—wear intensity, mm^3^/mm^2^,
*X*—average of the abrasive grain size, μm.

The negative quadratic coefficient in Equations (2) and (3) indicates a decrease in the growth rate of wear intensity at higher sandpaper granulations, which is also visible from Figure 11 and indicates the existence of a critical abrasive grain size.

## 4. Conclusions

In this paper, the abrasive wear resistance of two wear-resistant steels, Hardox 450 and XAR 450, was investigated. Abrasive wear was tested using two different techniques, the standard “dry sand—rubber wheel” method and by a Taber abrader device, with varying abrasive grain sizes of sandpapers. Based on the measured hardness values and microstructure analysis of the examined steels and the wear results, the following conclusions can be drawn:

(i) Both steels have high hardness, approximately 450 HV2, measured on all three characteristic sections and meet the hardness class 450,
(ii) The high hardness of wear-resistant steels is the result of the presence of the fine-grained martensitic microstructure,
(iii) Hardox 450 steel shows slightly higher wear resistance for both tested methods, which makes it a more appropriate choice for the manufacture of machine components exposed to intensive abrasive wear,
(iv) The wear regression equations allow for a reliable assessment of the wear intensity concerning the abrasive grain size. These results can serve as a guideline for steel selection in abrasive wear applications with different sizes of particles,
(v) A critical abrasive grain size was observed, between 21.8 and 30.2 μm, after which wear intensity does not change with an increase in abrasive grain size.

Future research will include a more extensive analysis of the microstructure and morphology of the worn surface by electron microscopy and X-ray diffraction, with a detailed consideration of abrasive wear micromechanisms at various abrasive sizes and different impact angles of abrasive particles.

## Figures and Tables

**Table 1:** Chemical composition of Hardox 450 and XAR 450 steel.

	Hardox 450		XAR 450	
Element	MD	OR	MD	OR
C	≤0.26	0.18 ± 0.010	≤0.22	0.17 ± 0.010
Mn	≤0.70	1.03 ± 0.011	≤0.80	0.97 ± 0.004
Si	≤1.60	0.26 ± 0.003	≤1.50	0.23 ± 0.002
P	≤0.025	0.007 ± 0.010	≤0.020	0.007 ± 0.003
S	≤0.010	0.002 ± 0.000	≤0.010	0.001 ± 0.001
Cr	≤1.400	0.001 ± 0.001	≤1.300	0.188 ± 0.001
Ni	≤1.500	0.024 ± 0.000	≤1.500	0.001 ± 0.000
Mo	≤0.60	0.02 ± 0.001	≤0.50	0.01 ± 0.001
B	≤0.005	0.001 ± 0.000	≤0.005	0.001 ± 0.000
Fe	balance	Balance	balance	balance

**Table 2:** Mass loss of samples by the “dry sand—rubber wheel” method.

Sample	Mass Loss, g	
	HARDOX 450	XAR 450
1	0.7728	1.6555
2	1.1608	1.5091
3	1.2169	1.5045
4	1.5655	1.3733
5	1.6340	1.2642
6	1.3735	1.4571
7	1.2493	1.4773
8	1.4444	1.4227
9	0.9316	1.0347
10	1.3577	0.9966
Mean value	1.2707	1.3695
Standard deviation	0.2541	0.2010
Coefficient of variation	20%	15%

**Table 3:** Mass loss of samples (mean values ± SD) after testing on the Taber abrader device.

Average Abrasive Grain Size, μm	Mass Loss, mg	
	HARDOX 450	XAR 450
15.3	1.60 ± 0.21	1.50 ± 0.56
21.8	6.78 ± 0.49	6.44 ± 0.36
30.2	8.50 ± 0.56	8.48 ± 0.61
35.0	8.52 ± 0.45	8.84 ± 1.55
46.2	9.18 ± 0.79	8.82 ± 0.44

**Table 4:** Regression analysis of wear results on the Taber abrader device for Hardox 450 steel (DF—degrees of freedom, SS—sum of squares, MS—mean square).

**Regression Statistics**					
Multiple R	0.95476				
R^2^	0.91158				
Adjusted R^2^	0.90354				
Standard Error	0.00590				
Observations	25				
**ANOVA**					
	DF	SS	MS	F-value	Significance F
Regression	2	0.00790	0.00395	113.40006	2.584 × 10^−12^
Residual	22	0.00077	3.482 × 10^−5^		
Total	24	0.00866			
	Coefficients	Standard Error	t Stat	*p*-value	
Intercept	−0.06787	0.01000	−6.78405	8.124 × 10^−7^	
Grain size	0.00669	0.00070	9.50461	3.018 × 10^−9^	
Grain size^2^	−8.548 × 10^−5^	1.132 × 10^−5^	−7.55335	1.512 × 10^−7^	

**Table 5:** Regression analysis of wear results on the Taber abrader device for XAR 450 steel (DF—degrees of freedom, SS—sum of squares, MS—mean square).

**Regression Statistics**					
Multiple R	0.94530				
R^2^	0.89360				
Adjusted R^2^	0.88392				
Standard Error	0.00835				
Observations	25				
**ANOVA**					
	DF	SS	MS	F-value	Significance F
Regression	2	0.01289	0.00644	92.38004	1.979 × 10^−11^
Residual	22	0.00153	6.975 × 10^−5^		
Total	24	0.01442			
	Coefficients	Standard Error	t Stat	*p*-value	
Intercept	−0.09457	0.01416	−6.67915	1.028 × 10^−6^	
Grain size	0.00902	0.00099	9.05286	7.146 × 10^−9^	
Grain size^2^	−1.177 × 10^−4^	1.602 × 10^−5^	−7.34928	2.343 × 10^−7^	

## Data Availability

The original contributions presented in this study are included in the article. Further inquiries can be directed to the corresponding author.
